# Taxonomic Revision of the Asiatic Widespread Filmy Fern *Cephalomanes javanicum* (Hymenophyllaceae, Polypodiidae) Reveals More Species than Expected

**DOI:** 10.3390/plants14081213

**Published:** 2025-04-15

**Authors:** Ya-Nan Zhao, Camille Regnier, Elodie Boucheron-Dubuisson, Kunio Iwatsuki, Atsushi Ebihara, Sabine Hennequin, Jean-Yves Dubuisson

**Affiliations:** 1 Institut de Systématique, Evolution, Biodiversité (ISYEB), Sorbonne Université, MNHN, CNRS, EPHE-PSL, Université des Antilles, CP 39, 57 rue Cuvier, 75005 Paris, France; yanan.zhao@etu.sorbonne-universite.fr (Y.-N.Z.); cam.regn@gmail.com (C.R.); elodie.dubuisson@sorbonne-universite.fr (E.B.-D.); sabine.hennequin@sorbonne-universite.fr (S.H.); 2 Campus Pierre et Marie Curie, Sorbonne Université, Bât. A, 4 place Jussieu, 75005 Paris, France; 3 Kamoshida, Aoba-ku, Yokohama 227-0033, Japan; iwatsuki@spa.nifty.com; 4 Department of Botany, National Museum of Nature and Science, 4-1-1 Amakubo, Tsukuba 305-0005, Japan; ebihara@kahaku.go.jp

**Keywords:** Hymenophyllaceae, *Cephalomanes*, taxonomy, phylogeny, tropical Asia, Oceania

## Abstract

This study revises the taxonomy of *Cephalomanes javanicum* (Hymenophyllaceae), a filmy fern traditionally considered widespread across the Indomalayan and Australasian regions. Through a comprehensive analysis of the literature, type specimens, and herbarium collections, we clarify the taxonomic status of three recognized varieties: typical *C. javanicum*, *C. javanicum* var. *sumatranum*, and *C. javanicum* var. *asplenioides*. Morphological, morphometric, and molecular phylogenetic investigations reveal that these varieties represent distinct species rather than intraspecific variants. Additionally, we reassess *C. atrovirens*, a species often confused with *C. javanicum*, and confirm that its two previously recognized subspecies also warrant species status. Based on these findings, we propose the elevation of the *C. javanicum* varieties and *C. atrovirens* subspecies to full species rank, providing updated taxonomic treatments, synonymy lists, and new lectotypifications. These revisions contribute to a more accurate understanding of species diversity within *Cephalomanes* and have broader implications for fern taxonomy, biogeography, and conservation in the tropical Asia, Australasian, and Oceanian regions.

## 1. Introduction

Hymenophyllaceae or filmy ferns, predominantly found in tropical and subtropical wet regions, are a highly diverse family of ferns encompassing over 600 species [1]. This family is divided into two monophyletic lineages, Hymenophylloideae and Trichomanoideae, which are primarily distinguished by their sori morphology. Mostly possessing tubular-based sori, the Trichomanoideae show significantly high diversity in other morphological traits. Therefore, various authors have proposed multiple generic classifications since the 19th century [2,3], with eight distinct genera currently recognized within Trichomanoideae [1,4]. Presl [2] described the new genus *Cephalomanes* based on his Asian species *Cephalomanes atrovirens* C.Presl, which presents simply pinnate fronds, differing from other Asian Hymenophyllaceae species described at the time. Later, Copeland [3,5] reused the genus *Cephalomanes* to define his *Trichomanes javanicum* group, which also exhibits simply pinnate fronds and is distributed from Madagascar to Polynesia. Despite some similarity and convergence with certain taxa of the distinct genus *Trichomanes* L. *sensu stricto* according to PPG I [4], such as the Malagasy *T. madagascariense* (Bosch) T.Moore that Copeland initially associated with his *T. javanicum* group, *Cephalomanes* species, as currently recognized [6], can still be differentiated by the following comprehensive features: linear-lanceolate to elliptic-linear fronds that are simply pinnate but with asymmetric pinnae and venation evidently anadromous; and campanulate sori arranged at the margin of pinnae, inducing more significant incisions along the edge. Many Neotropical species of Trichomanoideae have similar overall morphologies, but the majority show clearly catadromous venation and are genetically well integrated into the distinct genus *Trichomanes*, which also includes the anadromous species *T. madagascariense* [7]. The genus *Cephalomanes*, as considered here, is therefore restricted to Asia (the Indomalayan region), Australasia, and the Pacific islands.

According to the latest taxonomic revision [6], the genus *Cephalomanes* comprises five species: *C. atrovirens* as the type species, *C. crassum* (Copel.) M.G.Price, *C. densinervium* (Copel.) Copel., *C. javanicum* (Blume) C.Presl, and *C. singaporianum* Bosch. *Cephalomanes javanicum* is further divided into three varieties: *C. javanicum* var. *javanicum*, *C. javanicum* var. *asplenioides* (C.Presl) K.Iwats., and *C. javanicum* var. *sumatranum* (Alderw.) K.Iwats.

However, the taxonomy of the genus remains largely overlooked, and to date, no phylogenetic study has focused on the genus. This study falls within an ongoing review of the diversity of Hymenophyllaceae in China. In continental China and the main islands (Hainan and Taiwan), Flora of China [8] lists *C. javanicum* var. *sumatranum* in Hainan and *C. javanicum* var. *asplenioides* in Taiwan. It therefore seems important to ensure that the two varieties are unambiguous taxa. Varieties can in fact illustrate a high degree of infra-specific polymorphism, but the maintenance of infra-specific taxa must also reflect clearly discriminable and circumscribed groups/lineages and not obscure a continuous gradient of variability between extreme atypical forms. Additionally, several taxonomical issues arise, such as distinct species under the same voucher. For example, the sheet corresponding to voucher *Cuming 169* at the Edinburgh Herbarium contains individuals identified as *C. atrovirens* (E00413871, E00413872) on the left and top, with *C. javanicum* var. *asplenioides* (E00413870) on the bottom. These two species co-occur in certain regions, such as Luzon (Philippines), and the voucher appears to be a mixture of both taxa. Additionally, this voucher has been used as the type for the synonyms *Trichomanes rhomboideum* J.Sm. and *C. oblongifolium* C.Presl, with their taxonomic status becoming increasingly problematic. While Presl’s description and drawing [2] based on *Cuming 169* do clearly correspond to *C. atrovirens*, the status of synonyms remains to be clarified. A similar situation arises with *Cuming 184*, also collected in Luzon, which is considered the type for *C. javanicum* var. *asplenioides* and the synonym *T. curvatum* J.Sm. A re-examination of all the descriptions and specimens must therefore be undertaken, to formally clarify the identification of Chinese *C. javanicum*.

In this study, we therefore address the question of the taxonomy of *C. javanicum* and its varieties. In other words, do the different varieties define clearly distinct lineages? To answer this question, the present study combines a thorough taxonomic and nomenclatural work, morphological and morphometric studies, and molecular phylogeny. By incorporating Chinese specimens, our work refines taxon delimitation and clarifies synonymies, which will provide a stronger basis for future biogeographical and evolutionary research on the genus.

## 2. Materials and Methods

### 2.1. Taxon Sampling and Morphology

We focused here on *C. javanicum* and all associated names in IPNI [9] and Plants of the World Online—POWO [10], *Trichomanes javanicum* Blume, *T. sumatranum* Alderw., and *T. laciniatum* Roxb. would be three valid species names. Alternatively, Iwatsuki and Ebihara in *Flora Malesiana* [6] considered all three taxa as varieties of *C. javanicum*, with *T. laciniatum* treated as a synonym of *C. javanicum* var. *asplenioides*. In addition, Iwatsuki and Ebihara [6] adopted the generic treatment of PPG I [4], as we did here, contrary to POWO. These discrepancies led us to check all the relevant synonyms for these three taxa and their corresponding type specimens. As *C. atrovirens* has been found with *C. javanicum* under the same vouchers, resulting in confusion in collections, we also studied this second species in parallel and for comparison, also taking into account the two subspecies *C. atrovirens* subsp. *atrovirens* and *C. atrovirens* subsp. *boryanum* (Kunze) K.Iwats. recognized by Iwatsuki and Ebihara [6].

Finally, the investigated taxa of our study include *C. australicum* Bosch, *C. oblongifolium*, *C. wilkesii* Bosch, *T. acranthum* H.Itô, *T. acrosorum* Copel, *T. acrosorum* var. *alatum* Alderw., *T. alatum* Bory, *T. asplenioides*, *T. atrovirens*, *T. boryanum*, *T. curvatum*, *T. foersteri* Rosenst., *T. javanicum*, *T. javanicum* var. *intercalatum* Christ, *T. kingii* Copel., *T. laciniatum*, *T. ledermannii* Brause, *T. maluense* Brause, *T. preslii* C.V.Morton, *T. rhomboideum*, *T. rigidum* Wall., *T. setigerum* Wall., *T. suffrutex* Alderw., *T. sumatranum*, and *T. zollingeri* Bosch. Morphological studies were started with the review of type specimens and of morphological descriptions in original literature or from local floras [2,3,5,6,11,12,13,14,15,16,17,18,19,20,21,22,23,24,25,26,27,28,29,30,31,32,33]. All available type materials and representative specimens at B, BR, BRIT, CGE, E, F, GH, GOET, HUH, K, L, LE, MICH, MO, MSC, MU, MW, NY, PRC, S, US, UVMVT, W, WAG, and Z (herbarium acronyms following Thiers [34]) were consulted on JSTOR [35], on their official websites, or were provided by collaborators. Among them, types and/or specimens at BM, K, KUN, KYO, P, PE, TI, TNS, and YUKU were directly observed in herbaria. Investigations into specimens and collections also enabled us to verify and clarify geographical distributions of the diverse taxa. We adopted here an agnostic strategy, attempting to first assign each specimen to a morphological group, according to the criteria of Iwatsuki and Ebihara [6] and without considering a priori the specific identification of the specimen. This enabled us to reveal and correct a large number of misidentifications in the collections.

### 2.2. Morphometry

Following previous studies on other genera within the family [36], we conducted morphometric analyses based on frond measurements. Specifically, we measured the stipe length, lamina length, and frond width at its widest point for each specimen. To ensure consistency and minimize variability, all measurements were taken from the largest fertile frond of each specimen, following standardized protocols [36]. According to Iwatsuki and Ebihara [6], the position and distribution of sori on the fertile pinnae seem to be a key taxonomic criterion. To assess this, we measured the distances of both the most proximal and most distal sorus from the base of the pinna. The relative position of the sori was then quantified as a ratio of these distances to total pinna length, facilitating direct comparisons among groups. Measurements were taken per specimen on three randomly selected fertile pinnae, and the number of sori per selected pinna was also recorded.

To statistically assess the differences among taxa, we performed univariate non-parametric comparative analyses, accounting for differences in sample sizes among groups. The normality of each variable’s distribution was tested using the Shapiro–Wilk test, and variance homogeneity was assessed via Levene’s test. Depending on the results, appropriate multiple comparison tests were applied:

- Games–Howell for normally distributed data with unequal variances, in this case for lamina length;
- Kruskal–Wallis with Dunn’s post-hoc test for non-normally distributed data, in this case all other data.

All statistical analyses were performed using R version 4.3.2 [37] and RStudio v2024.12.0+467 [38], with a Bonferroni correction applied to account for multiple comparisons.

### 2.3. rbc*L* Phylogeny

Following the recent phylogenetic studies on the subfamily Trichomanoideae [7], we selected the single chloroplastic gene *rbc*L to resolve the intra-generic relationships of the *Cephalomanes* genus. The choice of this genetic marker ensures both sufficient phylogenetic resolution and data availability. We retrieved all sequences of *Cephalomanes* from the previous sequence dataset used by Dubuisson et al. [7] and added at least two new sequences of Chinese specimens: one identified as *C. javanicum* var. *asplenioides*, originated from Taiwan, while the other, identified as *C. sumatranum* from Hainan Island, was considered a variant of *javanicum* at that time. We retained it temporarily labeled as *C. javanicum* var. *sumatranum* in the sequence document. For the *C. javanicum* complex, sequences from Thailand and Borneo were also included, ensuring geographical diversity in the sampling. For *Cephalomanes atrovirens,* we included two specimens from Fiji and Micronesia representing the subspecies *boryanum* and one Japanese specimen representing the subspecies *atrovirens*. An *rbc*L sequence of *C. singaporianum* is available in GenBank (PP559978.1); however, a preliminary Blast search [39] and phylogenetic analyses revealed an unexpected position in the genus *Trichomanes s.s*. Although this result raises interesting biogeographical questions, we excluded this sequence of *C. singaporianum* from our sampling because it is outside the scope of this study.

The two remaining species of the genus are *C. crassum* and *C. densinervium*. *Cephalomanes crassum* has only been observed on the islands of Leyte and Samar (Philippines) and a single locality in North Sulawesi. This highly distinctive species shows a clear dimorphism between sterile and fertile fronds, which is not observed in any other species of the genus. It also differs in subsessile fronds or with very short stipes and deeply pinnatifid, whereas the other *Cephalomanes* species are clearly once-pinnate and have well-developed stipes. Its classification within *Cephalomanes* is based solely on the simply divided appearance of the fronds, raising doubts about this attribution. Unfortunately, *C. crassum* appears to be extremely rare or has been collected very rarely, with only a handful of historical herbarium specimens available and none that are suitable for molecular analysis. Field rediscovery is necessary to clarify its taxonomy. The second species, *C. densinervium*, restricted to eastern New Guinea and the Solomon Islands, also remains problematic. It closely resembles *C. atrovirens* subsp. *atrovirens* but, according to Iwatsuki and Ebihara [6], differs from it in that its stipes are much longer and the margins of the pinnae are much less cut or never lacerated, whereas margin lacerations are common in *C. atrovirens*. However, we did not manage to obtain the DNA sequence from available specimens.

We selected three species from each of the other seven genera of the Trichomanoideae subfamily as outgroups to confirm the monophyly of *Cephalomanes*. Additionally, four sequences from the *Hymenophyllum* genus were chosen as extra-groups to root the trees. Detailed voucher information and data sources can be found in Appendix A.

For DNA acquisition, *rbc*L amplification, sequencing, and analyses, we followed the protocol in Dubuisson et al. [7]. We employed the Bayesian inference (BI) approach for phylogenetic analysis on CIPRES Science Gateway v.3.3 [40]. Bayesian analysis was conducted with MrBayes 3.2.7 [41] using the GTR + G model, running four chains for 20 million generations with sampling every 1000 generations. Posterior probabilities were then calculated for evaluating the node support.

Additionally, we estimated the genetic divergence among taxa by computing uncorrected pairwise distances (*p*-distances) for *rbc*L within the ingroup. The *p*-distance was calculated by dividing the number of nucleotide differences (*n_d_*) by the total nucleotides compared (*n*), giving *p* = *n_d_*/*n*. All *p*-distances were calculated with PAUP version 4 [42].

## 3. Result

### 3.1. Morphology and Geography

**Figure 1:**
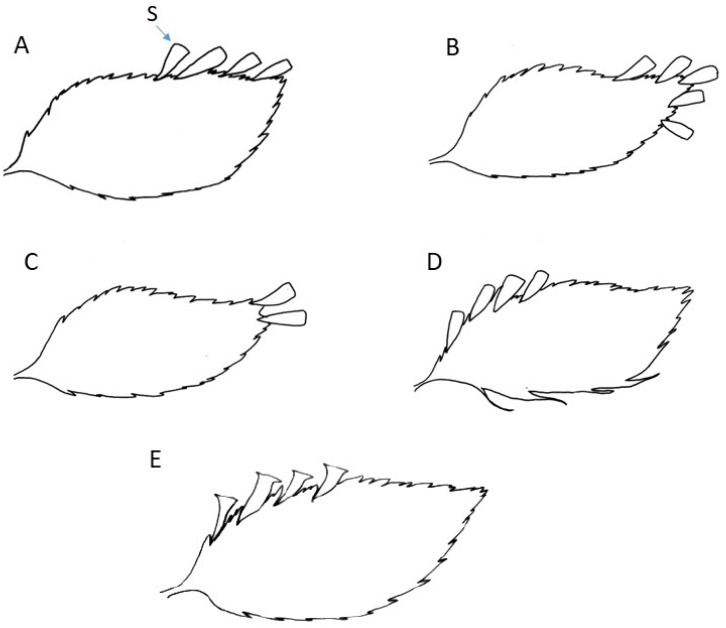
Comparison of fertile pinna according to the five morphological groups (see text), S = sorus: (**A**) *Cephalomanes javanicum* var. *javanicum*; (**B**) *C. javanicum* var. *asplenioides*; (**C**) *C. javanicum* var. *sumatranum*; (**D**) *C. atrovirens* subsp. *atrovirens*; (**E**) *C. atrovirens* subsp. *boryanum*. Drawings C. Regnier.

More than 313 specimens (including types) assignable to a morphological group were studied. It is evident that the taxa under consideration exhibit similar morphological characteristics, including a thick, erect rhizome densely covered with brownish hairs and bearing numerous robust roots. The fronds are clustered and erect, characterized by stiff stipes and sparse hairs analogous to those found on the rhizome. The lamina is consistently once-pinnate, with pinnae that are asymmetrically arranged, exhibiting a more or less falciform shape with margins that are either toothed or lacerate. Sori are typically located along the acroscopic margin of the pinnae, with sporangium receptacles (when mature) projecting from a truncate to dilated mouth. Nevertheless, we have identified subtle differences in the lamina shape and, notably, in the position of the sori (see Figure 1), which have prompted us to propose and subsequently confirm five distinct morphological groups.

**Figure 2:**
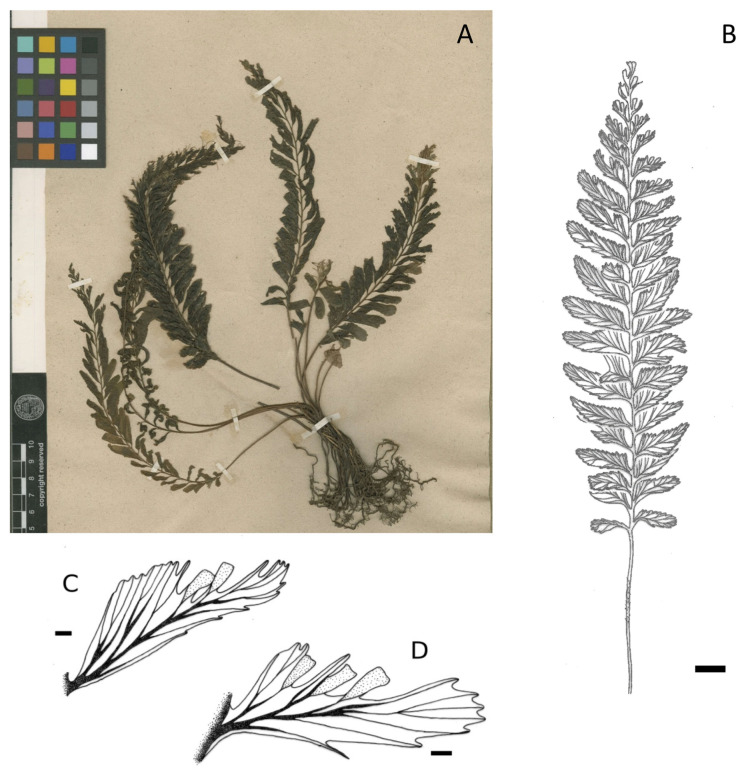
*Cephalomanes javanicum* var. *javanicum*: (**A**) Habitus, *C.L. Blume s.n.* (P00624413, isotype); (**B**) Frond, *K. Larsen et al. 41120* (P01474018)*,* scale = 1 cm; (**C**,**D**) Detail of fertile pinna, *K. Larsen* et al. *41120* (P01474017 and P01474018)*,* scale = 1 mm. Drawings C. Regnier.

**Group *C. javanicum* var. *javanicum*** (on 73 specimens observed): lamina elliptic-linear to linear, with 1 to 8(9) sori distributed only on the acroscopic side of the pinna, mostly in a distal position and usually on the most apical pinnae only, sometimes all over the upper margin, but rarely reaching the basiscopic side (Figure 1A and Figure 2).

**Figure 3:**
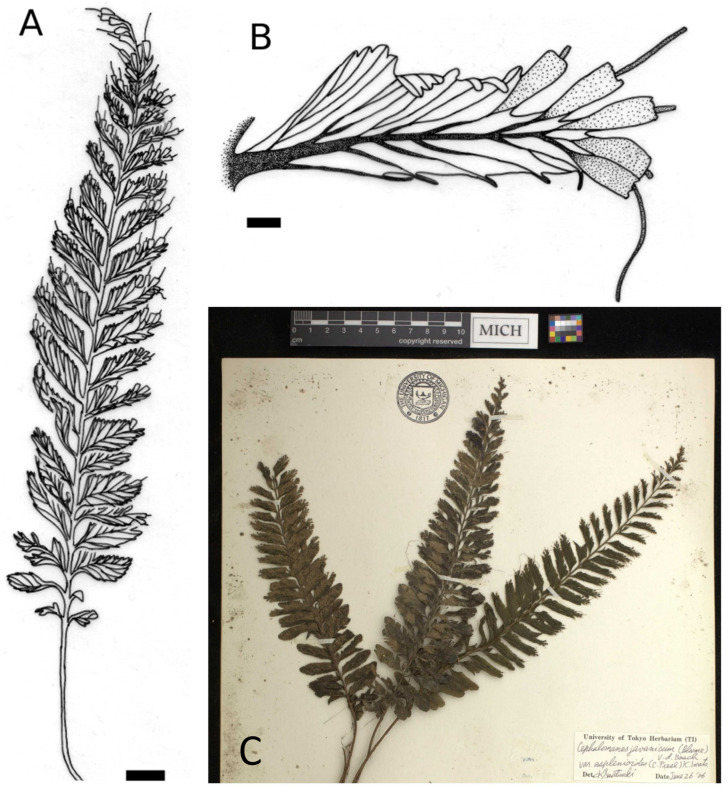
*Cephalomanes javanicum* var. *asplenioides*: (**A**) Frond, *E.B. Copeland 2053* (P01321990), scale = 1 cm; (**B**) Detail of fertile pinna, scale = 1 mm; (**C**) Habitus, *L.J. Brass 6620* (MICH1662070). Drawings C. Regnier.

**Group *C. javanicum* var. *asplenioides*** (128 specimens observed): lamina ovate-linear to linear-lanceolate, with 1 to 12 (rarely more) sori usually at the apex of pinnae not only on the acroscopic side but also on the basiscopic one and usually on the most apical pinnae, or on almost the whole frond for the more robust specimens (Figure 1B and Figure 3). This group may be easily confused with the variety *javanicum* because of the similar frond shape and their varied size.

**Figure 4:**
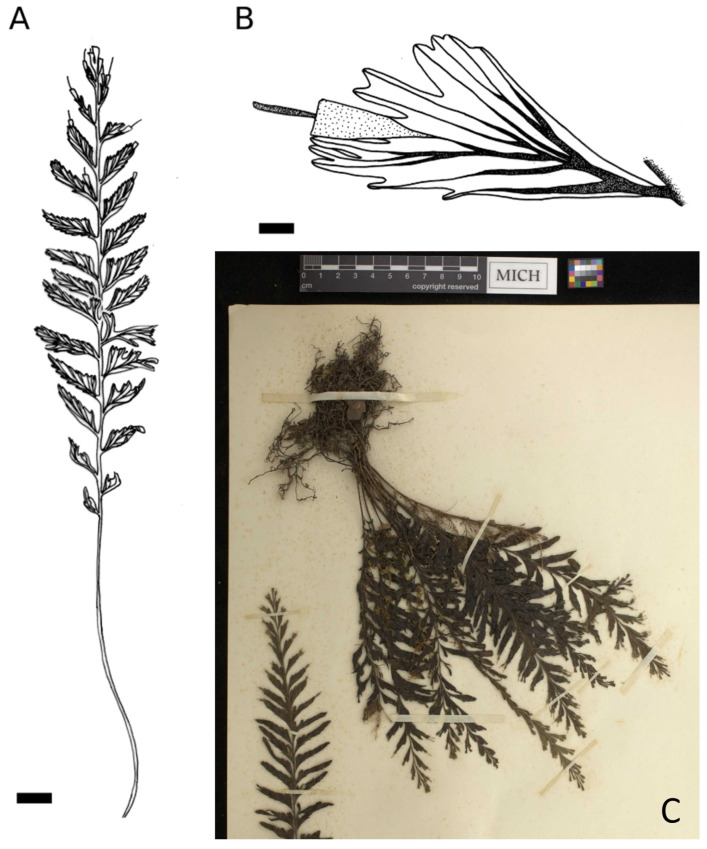
*Cephalomanes javanicum* var. *sumatranum*: (**A**) Frond, *s.c. s.n.* (P01627737)*,* scale = 1 cm; (**B**) Detail of fertile pinna, *E. Poilane 7017* (P01323066), scale = 1 mm; (**C**) Habitus, *H.H. Bartlett 7091* (MICH1662097). Drawings C. Regnier.

**Group *C. javanicum* var. *sumatranum*** (30 specimens observed): lamina elliptic-linear to linear or narrowly lanceolate, always with 1 to 3 sori (rarely more) strictly located at the apex of the pinna and usually on the most apical pinnae only (Figure 1C and Figure 4).

**Figure 5:**
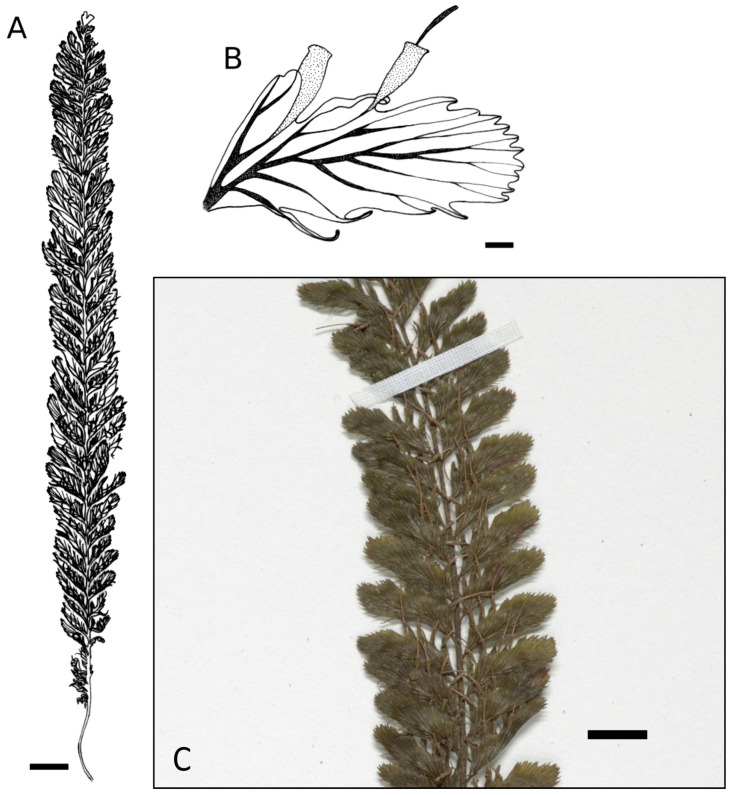
*Cephalomanes atrovirens* subsp. *atrovirens*: (**A**) Frond, *s.c. s.n.* (P01321968)*,* scale = 1 cm; (**B**) Detail of fertile pinna, *M. Ramos 39771* (P01331366)*,* scale = 1 mm; (**C**) Detail of fertile pinnae, *J.F. Barcelona et al. 2663* (US01406384), scale = 0.5 cm. Drawings C. Regnier.

**Group *C. atrovirens* subsp. *atrovirens*** (53 specimens observed): the most significant distinction of this group from the previous others lies in the fact that their 1 to 6 sori are exclusively distributed on the proximal portion of the upper margin of the pinna, with no distribution at the apex nor on the basiscopic side (Figure 1D and Figure 5). In addition, fertile pinnae can be seen from the lower third to the apex of the elliptic-linear to linear fronds and not always at the most apical positions.

**Figure 6:**
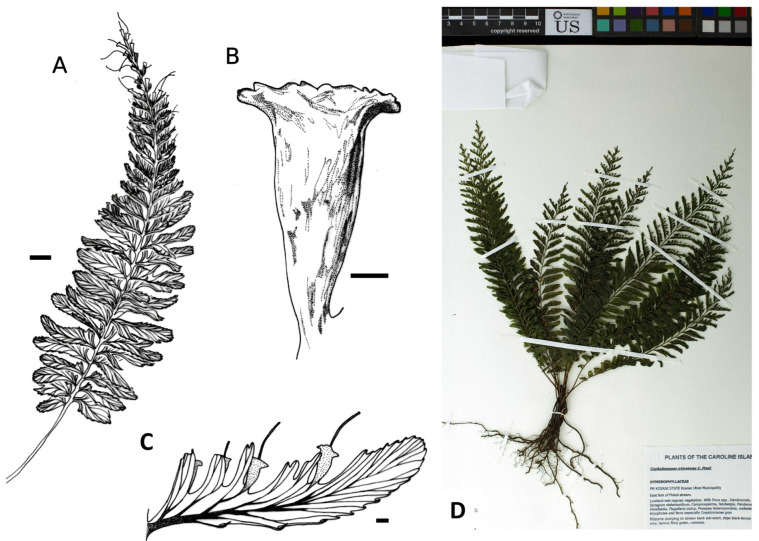
*Cephalomanes atrovirens* subsp. *boryanum*: (**A**) Frond, *M. Tuiwawa et al. 3043* (P01532886)*,* scale = 1 cm; (**B**) Detail of sorus with dilated lips*,* scale = 0.5 mm; (**C**) Detail of fertile pinnae, scale = 1 mm; (**D**) Habitus, *K.R. Wood 13676* (US01338425). Drawings C. Regnier.

**Group *C. atrovirens* subsp. *boryanum*** (27 specimens observed): lamina narrowly ovate to lanceolate, with 1 to 10 sori mostly on the proximal position on the acroscopic side, sometimes up to the apex, and mostly on the most apical pinnae only (Figure 1E and Figure 6). The group is particularly distinguished from the subspecies *atrovirens* group by its well-campanulate sori with normally well-dilated lips (Figure 6B,C). In other groups, the lips are usually truncate or slightly dilated.

**Figure 7:**
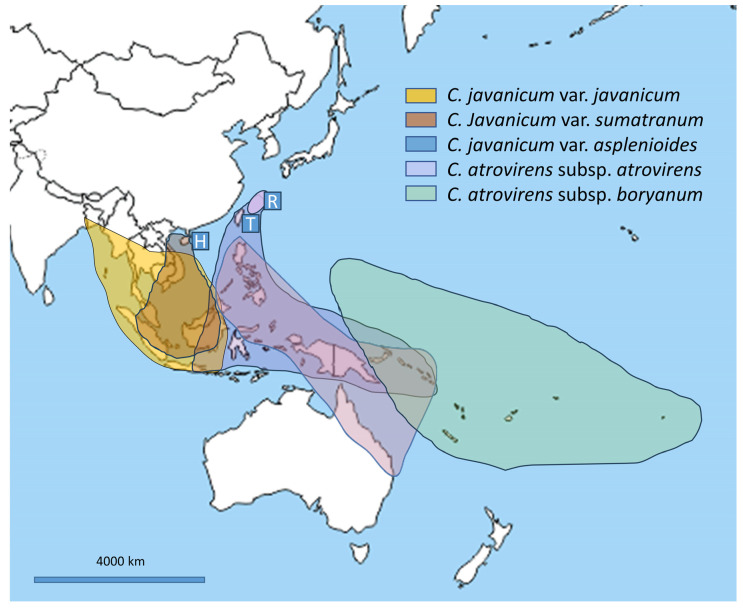
Geographical distribution of the three varieties assigned to *Cephalomanes javanicum* and of the two subspecies of *C. atrovirens*. H = Hainan, T = Taiwan, R = Ryukyu Islands (Japan). Free map background from d-maps (“https://d-maps.com/”, accessed on 1 September 2024).

Comparisons of other morphological characteristics and geographical distributions can be found in Table 1 and Figure 2, Figure 3, Figure 4, Figure 5, Figure 6 and Figure 7. The list of all specimens observed is available on request.

The *C. javanicum* var. *javanicum* group is distributed from eastern India, to Malaysia, western Indonesia (including Java), and Borneo. The *C. javanicum* var. *asplenioides* group expands northward to Japan (Ryukyu Islands), southward to Java, and eastward to Solomon Islands. The *C. javanicum* var. *sumatranum* group is found in Hainan (China) and Vietnam and expands southward to Sumatra and Borneo. The *C. atrovirens* subsp. *atrovirens* group has an eastern, wider distribution from Ryukyu Islands and the Philippines to Solomon Islands (and including northern Australia). The distribution of the *C. atrovirens* subsp. *boryanum* group does overlap with that of the typical *atrovirens* only in Bismarck and Solomon Islands, being restricted to Micronesia, eastern Melanesia, and Polynesia. Areas of distribution are overlapping between the groups of *C. javanicum* var. *javanicum* and *C. javanicum* var. *sumatranum* and between the groups of *C. javanicum* var. *asplenioides* and *C. atrovirens* subsp. *atrovirens*. *Cephalomanes javanicum* var. *javanicum* and *C. javanicum* var. *asplenoides* appear to be geographically isolated, except in few areas, and *C. atrovirens* subsp. *boryanum* appears to be a typical Oceanian taxon and the only taxon of the genus distributed east of the Solomon Islands.

### 3.2. Quantitative Data

All the descriptive statistics and results of the multiple comparison tests of means are reported in Table 2.

**Figure 8:**
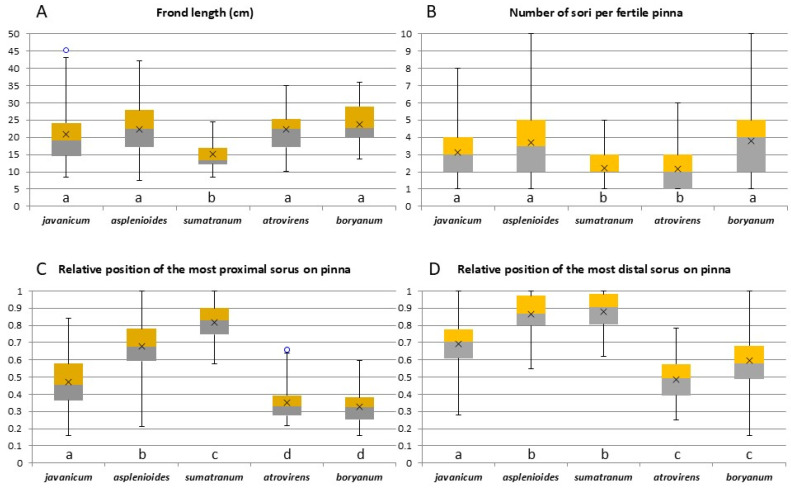
Distribution of some representative quantitative data per morphological group (as box and whisker plots, the separation between the grey and yellow boxes indicating the median value); *javanicum* = *C. javanicum* var. *javanicum*, *asplenioides* = *C. javanicum* var. *asplenioides, sumatranum* = *C. javanicum* var. *sumatranum*, *atrovirens* = *C. atrovirens* subsp. *atrovirens*, *boryanum* = *C. atrovirens* subsp. *boryanum*. (**A**) Frond length (cm); (**B**) Number of sori per fertile pinna; (**C**) Relative position of the most proximal sorus; (**D**) Relative position of the most distal sorus. The relative position is calculated as the ratio between the distance of the sorus from the pinna base and the whole pinna length (see text). The measures (with the units of measure if appropriate) on the Y axes are indicated in the subtitles and detailed above. Per comparison, a different letter indicates a significant difference (*p* < 0.05) with a Bonferroni correction (see Table 2).

*Cephalomanes javanicum* var. *sumatranum* is significantly smaller than the other taxa in terms of stipe length, lamina length, lamina width, and total frond length (Figure 8A). *Cephalomanes atrovirens* subsp. *boryanum* is the largest taxon for all frond measures, but there are no significant differences with the other species except *C. javanicum* var. *sumatranum*. The ratio of lamina length to stipe length does not provide a clear distinction among the five groups.

The number of sori per fertile pinna varies considerably across taxa (Figure 8B). *Cephalomanes atrovirens* subsp. *atrovirens* has the lowest average sorus count, but this number does not significantly differ from that of *C. javanicum* var. *sumatranum*. The three remaining taxa exhibit significantly higher average sorus counts, with *C. atrovirens* subsp. *boryanum* having the highest mean, although the differences among these three taxa are not statistically significant.

The two *C. atrovirens* subspecies have the lowest average relative positions of the most proximal sori. The subspecies *boryanum* has the lowest mean, but the differences are not significant between the two taxa (Figure 8C). The other three taxa have significantly higher mean values, all of which are significantly different, with the highest mean for *C. javanicum* var. *sumatranum*.

The two *C. atrovirens* subspecies exhibit the lowest mean relative positions of the most distal sori, with subsp. *atrovirens* having the lowest mean. However, the difference between the two taxa is not significant (Figure 8D). The other three taxa have significantly higher mean values. *Cephalomanes javanicum* var. *sumatranum* has the highest mean, but it does not significantly differ from that of *C. javanicum* var. *asplenioides*.

### 3.3. rbc*L* Phylogeny

**Figure 9:**
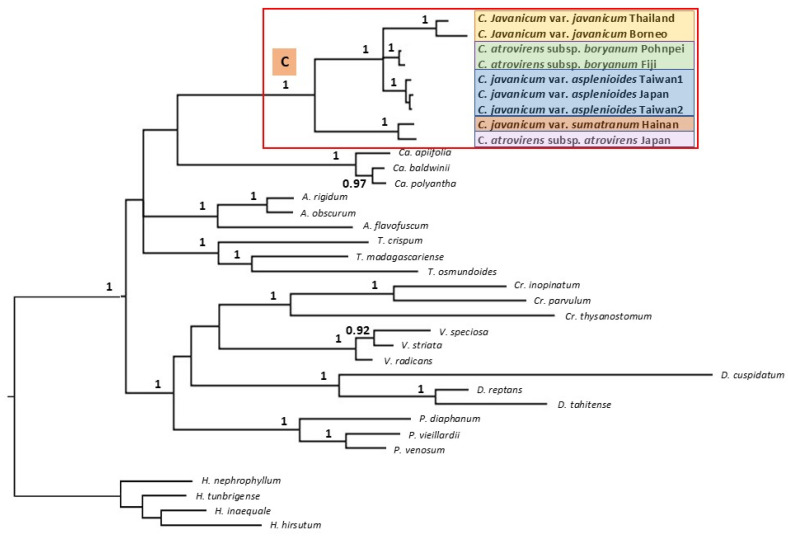
Bayesian inference (BI) phylogenetic tree including the five defined morphological groups of *Cephalomanes* (see text) based on *rbc*L sequences. The colors are those used in Figure 7. Posterior probabilities (>0.9) are shown on nodes. C = genus *Cephalomanes*, Ca = *Callistopteris*, A = *Abrodictyum*, T = *Trichomanes*, Cr = *Crepidomanes*, V = *Vandenboschia*, D = *Didymoglossum*, P = *Polyphlebium*, H = *Hymenophyllum*.

The phylogenetic analysis based on the *rbc*L gene revealed the paraphyly of both *C*. *javanicum* and *C. atrovirens* species as traditionally defined (Figure 9). *Cephalomanes javanicum* var. *sumatranum* and *C. atrovirens* subsp. *atrovirens* define a distinct lineage, while the other two varieties of *C. javanicum* clustered together with *C. atrovirens* subsp. *boryanum*, with strong support (posterior probability or PP = 1). Within the latter clade, each group delimits a distinct robust clade (PP = 1), but precise relationships among the three sub-clades remain unresolved.

## 4. Discussion

### 4.1. Morphological Groups

The first three groups, as detailed above, illustrate the three varieties of *C. javanicum* and could therefore reflect a high level of intraspecific variability, especially concerning frond measures. Similar to *C. atrovirens*, these varieties are often observed as rheophilous plants [6,33], thriving in very heterogeneous, even restrictive environments, which may explain the morphological intraspecific variation and high phenotypic plasticity. However, we observed very few ambiguous or intermediate individuals among our 313 studied specimens. Furthermore, the three *javanicum* varieties are fairly well differentiated and, as detailed above, show a homogeneous geographical distribution (see Figure 7).

### 4.2. Ambiguous Specimens and Synonyms

Although rare, a few problematic individuals remain, warranting closer examination. *Trichomanes foersteri* Rosenst., currently considered a synonym of *C. javanicum* var. *javanicum* [6], was described in 1914 [26] on two specimens from West Sumatra (holotype *l.W. Grashoff No.43*, S-P-5246). The sheet shows two entire specimens and an isolated robust frond. Both entire specimens have the morphology and sori typical of the *javanicum* variety. The isolated single frond, on the other hand, has some pinnae with clearly basiscopic sori. This last criterion would therefore assign the type of *T. foersteri* to the *asplenioides* variety, and *T. foersteri* would therefore be synonymous with the latter. However, the locality (western Sumatra) is incompatible with the supposed distribution of the *asplenioides* variety, leading to three possible explanations. The first would be that the isolated frond comes from a separate specimen and that its presence on the sheet is a mistake. However, in the absence of arguments to this effect, this remains highly speculative. The second hypothesis would suggest a rare case of an *asplenioides* variety dispersal far from its normal distribution area. The third and the most plausible is that the frond with basiscopic sori is an atypical variation within the *javanicum* variety. Thus, basiscopic sori alone would be insufficient for diagnosing the *asplenioides* variety, and some specimens will remain difficult to classify.

Another case concerns *Cephalomanes zollingeri* Bosch, currently treated as a synonym of the *javanicum* variety [6]. The species was described in 1857 [19] on Javanese specimens collected in 1848 by Zollinger (lectotype L.0544657, designated by Iwatsuki in 1991 [33]; isolectotypes: P00624411, P00624412, P00624443, BR0000013094959, and BR0000013094942). The lectotype, present at the Leiden (L) herbarium, indeed shows a robust specimen with sori that appear basiscopic, but the resolution of the scan available online does not allow this observation to be properly validated. In contrast, all but one isolectotype are relatively small, with sori positioned apically, aligning them more closely with *C. javanicum* var. *sumatranum*. In fact, only the observation of an isolectotype present at the Paris herbarium (P00624443) allows an unambiguous observation of fertile fronds with clearly basiscopic sori on many pinnae. *Cephalomanes zollingeri* would therefore be synonymous with the *asplenioides* variety. This highlights the challenge of identifying small or underdeveloped individuals.

As first highlighted by Copeland [5], *Cephalomanes* individuals can produce sori fairly early in their growth, before fronds reach their mature size and shape. In small, supposedly young individuals, this results in sori borne on the most apical pinnae, which are often small, making it difficult to determine their relative position. As defined by Iwatsuki [33], we therefore have forms with sori on apical spike-like, sometimes lacking lamina, structures. This corresponds, for example, to the *kingii* and *acrosorum* (as the epithet indicates) forms of the *C. atrovirens* species [6,33], both known from only a few specimens in New Guinea. Given their rarity and highly atypical morphology, we have not included them in our morphometric analyses, and they cannot be used molecularly.

Similarly, *Trichomanes acranthum* H.Itô was proposed for populations in the Ryukyu Islands and represents the northernmost variant of *C. atrovirens*. Individuals are generally small and, as expected, have sori restricted to small apical pinnae and can reach the pinna apex, contrary to typical *C. atrovirens*. More developed specimens (e.g., TNS01176791) exhibit proximal sori and slightly dilated lips, supporting their placement within *C. atrovirens*, yet their fertile pinnae are more restricted in distribution than in larger, more robust individuals. As with the *kingii* and *acrosorum* forms, the “*acranthum*” form was not included in the morphometric analyses, but we have decided to include it in the updated key proposed below.

### 4.3. Cephalomanes atrovirens Taxonomy

*Cephalomanes atrovirens* subsp. *boryanum* is based on *Trichomanes boryanum* described by Kunze [17] to designate Oceanian populations distinct from the *C. javanicum* and *C. atrovirens* species. Iwatsuki [33] attributed the taxon to *C. atrovirens* on the basis of the mostly proximal position of the sori on the pinnae and to a distinct subspecies on the basis of its geographical distribution and in particular the well-dilated lips of the sori. Our analyses further indicate that *boryanum* subspecies has significantly wider fronds and a higher average number of sori than *atrovirens* subspecies, with sori sometimes extending to the pinna apex. Additionally, its fertile pinnae are mostly restricted to the frond apex, unlike *atrovirens* subspecies, in which they are more evenly distributed. These criteria alone do not contradict a geographic subspecies status for the *boryanum* taxon, but the phylogenetic position clearly indicates a specific taxon distinct from typical *C. atrovirens*. Furthermore, as Braithwaite [32] points out, the typical *C. atrovirens* is characterized by a chromosome number of *n* = 32, whereas specimens identified as *boryanum* subspecies have a chromosome number of *n* = 64. This would support the distinction of two species. We here agree with POWO, which treats *boryanum* subspecies as a distinct valid species.

During our investigations of the herbarium material, we identified 19 individuals assigned to *C. densinervium* based on morphological characteristics. However, no material was available for the molecular analysis. Statistical analysis of the quantitative data, not detailed here, failed to differentiate these specimens from *C. atrovirens* subsp. *atrovirens*, with the two taxa differing only in the average number of sori per fertile pinna. *Cephalomanes densinervium* may be a form or variety of *C. atrovirens* whose less toothed or unlacerated margins could reflect less rheophilic ecological preferences than typical *C. atrovirens* (laceration of the pinnae could make the fronds less resistant to water currents). Given the lack of genetic data, we refrain from further discussing its taxonomic status here.

### 4.4. Importance of Sorus Characteristics

Traditionally, sorus position has been a key qualitative criterion for species delimitation within the genus [6]. However, its subjectivity poses challenges, particularly for ambiguous specimens with intermediate or overlapping traits. This is illustrated by the high variance in the quantitative data including frond measures (see Figure 8B–D). Nevertheless, statistical tests, in particular on the relative position of the sori, enabled the five groups to be more or less well isolated. It should be noted, however, that each quantitative trait alone cannot be used to assign a specimen to a group. For example, many *C. javanicum* var. *javanicum* specimens may have their most proximal sori in a more distal position than the most proximal sori of some *C. javanicum* var. *asplenioides* specimens, and this is due to the high variance in each of the data. Consequently, species identification must rely on a combination of sorus characteristics and geographical distribution.

### 4.5. Species Delineation

Morphological and phylogenetic analyses support the recognition of *C. sumatranum* as a distinct species, based primarily on its unique sorus positioning and smaller average frond size. *Cephalomanes sumatranum* is genetically associated with *C. atrovirens* subsp. *atrovirens*, but both taxa are clearly distinguished by both morphology and sori characteristics. However, we should point out here that the sequenced *C. atrovirens* specimen comes from the Ryukyu Islands and in fact corresponds to the atypical “*acranthum*” form (see above), which in any case is also morphologically very different and geographically very distant from *C. sumatranum*. We here consider that the “*acranthum*” specimen is representative of the taxon *C. atrovirens*. Future studies should include additional sequences from typical *C. atrovirens* specimens to further clarify species boundaries.

Then, for the clade including the other three morphological groups, we also suggest defining three distinct taxa. Firstly, typical *C. javanicum* and *C. atrovirens* subsp. *boryanum* would belong to two undisputed distinct specific taxa, each with clearly distinguishable morphological traits and displaying significantly different distributions, suggesting geographic isolation. Secondly, the differences between *C. javanicum* var. *asplenioides* and *C. atrovirens* subsp. *boryanum*, which have an overlapping distribution, are also notable and concern the arrangement of the sori and the shape of the sorus lips. Finally, the typical *C. javanicum* and *C. javanicum* var. *asplenioides* have largely disjoint distributions, with *asplenioides* being the only variety whose sori can be mostly found on the basiscopic margin.

Phylogenetic tree topology alone does not fully resolve the relationships among *C. javanicum*, *C. javanicum* var. *asplenioides*, and *C. atrovirens* subsp. *boryanum*. Therefore, maintaining *asplenioides* as a variety of *C. javanicum* remains a possibility. To resolve this, additional markers (such as faster-evolving inter-genes) would be necessary. However, our current data allow us to propose taxonomic hypotheses. Genetic distances based on *rbc*L (*p*-distance; Table 3) further support species-level separation: the average distance between *C. javanicum* and *C. javanicum* var. *asplenioides* is 0.022, equivalent to the average distance between *C. javanicum* and *C. atrovirens* subsp. *boryanum* (0.021) and significantly greater than the average distance between *C. javanicum* var. *asplenioides* and *C. atrovirens* subsp. *boryanum* (0.009). For comparison, the average distance between *C. sumatranum* and all other taxa (except typical *C. atrovirens*) is 0.042, that between *C. atrovirens* and all other taxa (except *C. sumatranum*) is 0.041, and that between *C. atrovirens* and *C. sumatranum* is 0.008. This would support the hypothesis of three distinct species in the clade associating C. *javanicum*, *C. javanicum* var. *asplenioides*, and *C. atrovirens* subsp. *boryanum*. It would be useful to compare these values with an established species-level genetic divergence threshold, such as the Average Genetic Distances of Sister Species (AGDS) proposed for angiosperms, particularly based on ITS2 [43]. However, no equivalent AGDS threshold currently exists for ferns (specifically for *rbc*L), and applying values derived from seed plants would be inappropriate. In our case, the AGDS within the genus would vary from 0.008 to 0.022. By way of comparison, within the distinct trichomanoid genus *Polyphlebium* Bosch, the average genetic distance between species has been estimated at 0.034 [44]. There is therefore a very high degree of variability within the Trichomanoideae lineage itself, which makes it difficult to set a reference threshold. The values calculated here are thus comparable only at the scale of the group in question.

### 4.6. Taxonomic Treatment

#### 4.6.1. Nomenclature

*Cephalomanes javanicum* var. *asplenioides* is based on *C. asplenioides* C.Presl published in 1848 [18], a name supposedly valid based on *Trichomanes asplenioides* C.Presl published in 1843 [2]. However, the latter was illegitimate, as Swartz [45] had already used the same binomial for a distinct Neotropical species. Moreover, the material used by Presl (*Cuming 184*) had also been used in 1841 for the publication of *T. curvatum* J.Sm. [14], which is in fact a *nom. nud.* in the absence of a valid description and therefore a homotypic synonym.

*Trichomanes laciniatum* Roxb. was published in 1844 [15] on specimens from the Mollucas (BR0000006987763), which unambiguously exhibit *C. asplenioides* morphology. This species would therefore be the valid taxon and would invalidate Presl’s *C. asplenioides*, which would then be a heterotypic synonym. On this basis, POWO recognizes *T. laciniatum* as valid, and following the PPG system [4], we adopt its combination under *Cephalomanes*, designating it as *C. laciniatum* (Roxb.) De Vol. Our findings also confirm Morton’s [30] nomenclatural treatment of these taxa. However, in the same paper, Morton designates a ‘holotype’ in the Prague herbarium (acronym PRC) for *C. asplenioides*, specifying *Cuming 169*. In contrast, Presl had clearly based both *T. asplenioides* and *C. asplenioides* on *Cuming 184* [2,18]. Morton therefore quotes *Cuming 184* for *T. asplenioides* but *Cuming 169* for *C. asplenioides*, which is a mistake. In fact, the only Cuming’s specimen seen at PRC is indeed a *Cuming 169* (PRC450314), but it is the type of *C. oblongifolium* [6,33]. Morton based his choice on Holttum’s photograph, which is thus probably that of the PRC type of *C. oblongifolium*. The same photograph is available at Kew (K000375596) and confirms this. We therefore invalidate Morton’s “lectotypification” and hereafter designate a new lectotype for *T. asplenioides* based on a *Cuming 184* specimen.

Additionally, *T. rhomboideum* published in 1841 [14] and based on *Cuming 169* is also a *nom. nud.* due to the lack of description. Based on the form I of its type specimen *Cuming 169*, *C. atrovirens* was published in 1843 [2]. Then, *C. atrovirens* was combined under *Trichomanes* by Kunze [16], indicating *T. rhomboideum* as the synonym. This was the first time John Smith’s specific epithet “*rhomboideum*” had been treated since its publication. Afterwards, *C. oblongifolium* was published in 1848 [18] based on *Cuming 169* form II, which is considered a form of *C. asplenioides*. The type specimen of *T. rhomboideum* (K000375661) clearly exhibits *C. asplenioides* features too, which suggests that we should consider the two taxa as synonyms of *C. laciniatum*. *Cuming 169* is clearly a mixture of the two species *C. atrovirens* and *C. laciniatum*. In the detailed treatment below, we will specify to which species all the *Cuming 169* specimens we have been able to study should be assigned. In addition, apart from one ambiguous specimen from the Leiden herbarium (L.0544639), *Cuming 184* individuals seem to group only specimens of *C. laciniatum*.

Consequently, we retained the following five species names: *C. javanicum* (Blume) C.Presl, *C. laciniatum* (Roxb.) De Vol, *C. sumatranum* (Alderw.) Copel., *C. atrovirens* C.Presl, and *C. boryanum* (Kunze) Bosch.

This revision aligns with POWO’s recognition of *T. boryanum*, *T. laciniatum*, and *T. sumatranum* while integrating them into *Cephalomanes* based on PPG conventions. In fact, POWO seems to adopt the list of species proposed by Copeland in 1938 [3], who also treated the *kingii* and *acrosorum* forms of *C. atrovirens* as distinct species. However, POWO does not incorporate Iwatsuki’s [33] more recent revision, which recognized three varieties of *C. javanicum* and two subspecies of *C. atrovirens*. Iwatsuki’s classification suggested that these taxa represented highly variable, widely distributed species with well-defined geographical and morphological subspecific units. The monophyly of each species was thus expected, and it would have left the primary discussion centered on whether eventual inferred subclades warranted subspecific recognition. Unexpectedly, our study reveals the polyphyly of these species, necessitating a reassessment of the numerous taxa described since the 19th century. The five species recognized here are primarily supported by genetic data, with additional differentiation based on sorus characteristics and geographic distribution. For this reason, we propose below a new key that also includes the species not selected for phylogenetic analysis.

#### 4.6.2. Key of the Studied Species and New Treatment

Based on the key to species of *Cephalomanes* used by Iwatsuki and Ebihara [6] and corrected with the present work, we propose here an updated key that designates *C. sumatranum* and *C. laciniatum* (=*C. asplenioides*) as two distinct species from *C. javanicum*, and *C. boryanum* as distinct from *C. atrovirens*.

1a. Fronds monomorphic and always with distinct pinnae; stipes always developed.2b. Fronds dimorphic, usually subsessile or with short stipes, and usually deeply pinnatifid.
*C. crassum*
2a. Sori always distributed on the most apical pinnae, usually never from the first third of the frond or on the whole frond.5b. Sori often distributed from the first third of the frond to the apex, sometimes on the whole frond.33a. Sori always on the margin of the pinnae in the acroscopic and proximal positions.4b. Sori usually acroscopic, reaching the apex of the pinnae, and often present on the basiscopic side.
*C. laciniatum*
4a. Lamina margins usually not lacerated, maximum slightly toothed; stipes reaching 20 cm long; absent west of New Guinea and in Australia.
*C. densinervium*
b. Lamina margins often lacerated or deeply toothed; stipes usually not exceeding 13 cm long; present from eastern Borneo to Solomon Islands and Australia (Queensland).
*C. atrovirens*
5a. Sori always present at the apex of the pinnae and never in proximal positions.6b. Sori normally distributed from the base to the apex of the pinnae.76a. Sori 1 to 12 (more often 4–5), usually not exceeding 3.5 mm long, often present on the basiscopic margin, and not strictly restricted to the apex.
*C. laciniatum*
b. Sori 1 to 3 (rarely more), reaching 4.5 mm long, strictly apical, never on the basiscopic margin.
*C. sumatranum*
7a. Sori sometimes or mostly in proximal positions, absent from Japan.8b. Sori usually in proximal positions; restricted to Japan (Ryukyu Islands).*C. atrovirens* (*“acranthum”* form)8a. Sori mostly in proximal positions, always with well-dilated lips; distributed in Micronesia and from Bismarck Islands to Polynesia.
*C. boryanum*
b. Sori sometimes in proximal position but more often in distal ones; with truncate to slightly dilated lips; distributed from eastern India to Borneo.
*C. javanicum*


***Cephalomanes javanicum*** (Blume) C.Presl, Abh. Königl. Böhm. Ges. Wiss., ser. 5, 5: 334 (1848)

Based on *Trichomanes javanicum* Blume, Enum. Pl. Javae 2: 224 (1828) ≡ *Lacostea javanica* (Blume) Prantl, Unters. Morph. Gefässkrypt.: 50 (1875). Type—Indonesia, Java, Nusa Kambangan Island, *s.d.*, *C.L*. *Blume s.n.* (holotype: L.0544656!; isotypes: P00624413!, P00624414!).

= *Trichomanes foersteri* Rosenst., Repert. Spec. Nov. Regni Veg. 13: 213 (1914). Type—Indonesia, Sumatra, Padang Panjang, Oct. 1913, *l.W. Grashoff No.43* (holotype: S-P-5246!). Note: An isolated frond shows basiscopic sori in some pinnae, an atypical case in *javanicum* (see discussion above).

= *Trichomanes rigidum* Wall., Numer. List: n.° 161 (1829), not validly published.

= *Trichomanes setigerum* Wall., Numer. List: n.° 158 (1829). Type—Myanmar, Chappedong, 1827, *N. Wallich 158* (K001109412!, K001090185!), not validly published.

Short description: Thick, short and erect rhizomes with robust roots and clustered erect fronds; fronds well stipitate and once-pinnate, up to 40 cm long, elliptic-linear to linear or narrowly lanceolate; up to 9 sori per pinna, usually distributed on the most apical pinnae and on the acroscopic margin from the base to the apex of the pinnae (rarely on the basiscopic side), with truncate to slightly dilated lips.

Distribution: Northeast India, Bangladesh, Myanmar, Andaman and Nicobar Islands, Thailand, South Vietnam (Annam), Peninsular Malaysia, Singapore, Sumatra, Java, Borneo.

***Cephalomanes laciniatum*** (Roxb.) De Vol, Fl. Taiwan 1: 98 (1975)

Based on *Trichomanes laciniatum* Roxb., Calcutta J. Nat. Hist. 4: 518 (1844). Type—Indonesia, Molucca Islands, *s.d.*, *C. Smith s.n.* (lectotype BR0000006987763!, designated by Morton, Contri. U.S. Natl. Herb. 38: 382 (1974)).

= *Cephalomanes asplenioides* C.Presl, Abh. Königl. Böhm. Ges. Wiss., ser. 5, 5: 334 (1848) ≡ *Trichomanes asplenioides* C.Presl, Hymenophyllaceae: 15, 37 (1843), non Sw., *nom. illeg*. ≡ *Cephalomanes javanicum* var. *asplenioides* (C.Presl) K.Iwats., J. Fac. Sci. Univ. Tokyo, Sect. 3, Bot. 13: 549 (1985). Type—Philippines, Luzon, *s.d.*, *Cuming 184 p.p.* (lectotype: P00624440!, here designated; isolectotypes: CGE00050063!, E00413868!, E00413869!, GH0022199!, GOET009199!, HUH00022199!, K000375660!, K000375462!, L.0544640!, P00624441!, P00624442!, US00134586!, WAG.1729893!). Note: The *Cuming 184* L.0544639 specimen shows a frond (right) exhibiting *C. atrovirens* sori and is thus excluded from the type list. Morton designates a PRC specimen as “holotype” [30], but it is a *Cuming 169* and thus a mistake (see discussion above).

= *Trichomanes curvatum* J.Sm., J. Bot. (Hooker) 3: 417 (1841), *nom. nud.* ≡ *Cephalomanes curvatum* (J.Sm.) Bosch, Ned. Kruidk. Arch. 4: 351 (1859). Based on the same type material as *T. asplenioides* C.Presl.

= *Trichomanes rhomboideum* J.Sm., J. Bot. (Hooker) 3: 417 (1841), *nom. nud.* Type—Philippines, Luzon, *s.d.*, *Cuming 169 p.p.* (lectotype: K000375661!, here designated; isolectotypes: CGE00050062!, K000375630!, MO100438391!).

= *Cephalomanes oblongifolium* C.Presl, Abh. Königl. Böhm. Ges. Wiss., ser. 5, 5: 334 (1848). Type—Philippines, Luzon, *s.d.*, *Cuming 169 p.p.* (lectotype: PRC450314!, designated by Iwatsuki [33]; isolectotypes: CGE00076425!, E00413870!, E00413873!, LE00007950!, MICH1190163!, P00624502!, P00624503!, PRC456183!—original Presl’s drawing, US00134616!, W0052720!).

*= Trichomanes zollingeri* Bosch, Pl. Jungh. [Miquel]: 552 (1857) ≡ *Cephalomanes zollingeri* (Bosch) Bosch, *Hymenophyll. Javan*. 31–32, pl. 23 (1861) ≡ *Lacostea zollingeri* (Bosch) Prantl, Unters. Morph. Gefässkrypt.: 50 (1875). Type—Indonesia, Java, 1848, *H. Zollinger 1464* (lectotype: L.0544657!, designated by Iwatsuki [33]; isolectotypes: P00624411!, P00624412!, P00624443!, BR0000013094959!, BR0000013094942!).

= *Trichomanes javanicum* var. *intercalatum* Christ, Philipp. J. Sci. C 2: 156 (1907). Type—Philippines, Luzon, Benguet, Sablan, Apr. 1904, *A.D.E. Elmer 6214* (lectotype: MICH1191075!, here designated); Province of Laguna, Mount Maquiling, Jan. 1906, *A. Loher s.n.* (remaining syntype not located); Province of Rizal, Oriud, Feb. 1906, *A. Loher s.n.* (remaining syntype not located).

= *Trichomanes ledermannii* Brause, Bot. Jahrb. Syst. 56: 35 (1920) ≡ *Cephalomanes ledermannii* (Brause) Copel., J. Arnold Arbor. 24: 440 (1943). Type—Indonesia, New Guinea, 11 Sep. 1912, *C.L. Ledermann 8638* (lectotype: B 20 0105007!, designated by Iwatsuki and Ebihara [6]); *C.L. Ledermann 9622* (remaining syntypes: B 20 0105008!, BM001044280!, NY00127568!). Note: Taxon initially considered synonymous with *C. atrovirens*. However, the small specimens have fertile pinnae only at the apex, with sori that do not seem to be predominantly proximal and some of which are apical, suggesting small forms of *C. asplenioides* that are also found in the region.

= *Trichomanes maluense* Brause, Bot. Jahrb. Syst. 56: 36 (1920). Type—Indonesia, New Guinea, Sepik River, 30 Mar. 1912, *C.L. Ledermann 6843* (lectotype: B 20 0105056!, designated by Iwatsuki [33]; isolectotypes: UC391776!). Note: Taxon with small specimens resembling those of *T. ledermannii* and observed in the same region; initially also considered to be synonymous with *C. atrovirens*. The presence of numerous pinnae with sori in basiscopic position (B 20 0105056) unambiguously assigns this taxon to *C. asplenioides* and would also support the status proposed here for *T. ledermannii*.

= *Trichomanes suffrutex* Alderw., Nova Guinea 14: 56 (1924). Type—Indonesia, New Guinea, Irian Jaya, Pionier Bivouac, 13 Sep. 1920, *H.J. Lam 1197* (lectotype: L.0544612!, here designated; isolectotypes: K000377094!, US00134627!); *H.J. Lam 1334* (remaining syntype not located).

= *Trichomanes preslii* C.V.Morton, Contr. U.S. Natl. Herb. 38: 190 (1968). Note: *nom. nov.* for *Trichomanes asplenioides* C.Presl that had been considered as a *nom. illeg*.

Short description: Thick, short, and erect rhizomes with robust roots and clustered erect fronds; fronds well stipitate and once-pinnate, up to 45 cm long, ovate-linear to linear-lanceolate; up to 12 sori per pinna, often distributed from the first third of the frond to the apex, sometimes on the whole frond, less often on the most apical pinnae, always present at the apex of the pinnae, and never in proximal positions, usually on the acroscopic margin and often present on the basiscopic side, with usually truncate lips.

Distribution: Ryukyu Islands (Japan), Taiwan, Philippines, eastern Borneo, Java, Sulawesi, Moluccas, Papua, New Guinea, Bismarck and Solomon Islands.

***Cephalomanes sumatranum*** (Alderw.) Copel., Philipp. J. Sci. 67: 67 (1938)

Based on *Trichomanes sumatranum* Alderw., Bull. Dépt. Agric. Indes Néerl. 18: 4 (1908) ≡ *Cephalomanes javanicum* var. *sumatranum* (Alderw.) K.Iwats., J. Fac. Sci. Univ. Tokyo, Sect. 3, Bot. 13: 549 (1985). Type—Indonesia, Sumatra, *s.d.*, *Burck s.n.* (holotype: BO!).

Short description: Thick, short, and erect rhizomes with robust roots and clustered erect fronds; fronds well stipitate and once-pinnate, up to 30 cm long, elliptic-linear to linear or narrowly lanceolate; 1–3 (rarely more) sori per pinna, always on the most apical pinnae, strictly apical on the pinnae, never on the basiscopic margin, with usually truncate lips.

Distribution: China (Hainan), Vietnam, Borneo, Sumatra, to be checked for Peninsular Malaysia, Thailand, Laos, and Cambodia.

***Cephalomanes atrovirens*** C.Presl, Hymenophyllaceae: 18 (1843)

≡ *Trichomanes atrovirens* (C.Presl) Kunze, Bot. Zeitung (Berlin) 5: 371 (1847) ≡ *Trichomanes javanicum* var. *atrovirens* (C.Presl) C.Chr., Index Filic.: 635 (1906). Type—Philippines, Luzon, *s.d.*, *Cuming 169 p.p.* (lectotype: PRC456179!, here designated; isolectotypes: E00782202!, E00413871!, E00413872!, GH00020718!, GOET009200!, HUH00020718!, L.3517155!, L.3517156!, LE0007951!, MICH1190162!, MW0591065!, P00624501!, W0200683!, Z000002301!). Note: Croxall designated a specimen from Kew (K herbarium) as the lectotype [30], but this has not been found and is therefore presumed lost. In fact, all *Cuming 169* specimens available at K turned out to be *C. laciniatum*. We have therefore had to designate a new lectotype here.

= *Trichomanes acranthum* H.Itô, Iconogr. Pl. Asiae Orient. 2: 110, t. 45 (1937) ≡ *Cephalomanes acranthum* (H.Itô) Tagawa, Acta Phytotax. Geobot. 14: 45 (1950). Type—Japan, Ryukyu, Iriomote Island, 21 May 1936, *S. Sonohara and H. Itô s.n.* (holotype: TI00080886!).

= *Trichomanes kingii* Copel., Philipp. J. Sci., C 6: 72 (1911) ≡ *Cephalomanes kingii* (Copel.) Copel., Philipp. J. Sci. 67: 68 (1938) ≡ *Cephalomanes atrovirens* f. *kingii* (Copel.) K.Iwats., J. Jap. Bot. 66: 141 (1991). Type—New Guinea, Big Goia, *s.d.*, *Copland King S13* (holotype: MICH1191076!). Note: The taxon is only represented by a single small frond in poor condition with sori borne on apical segments or pinnae without lamina. Its assignment to the species *C. atrovirens* remains doubtful, and the taxon could also be an atypical *C. asplenioides*.

= *Trichomanes acrosorum* Copel., Philipp. J. Sci., C 6: 72 (1911) ≡ *Cephalomanes acrosorum* (Copel.) Copel., Philipp. J. Sci. 67: 68 (1938) ≡ *Cephalomanes atrovirens* f. *acrosorum* (Copel.) K.Iwats., J. Jap. Bot. 66: 141 (1991). Type—New Guinea, Lakekamu, Apr. 1910, *Copland King 352* (holotype: MICH1191059!). Note: The single small type specimen has sori on apical segments or pinnae without lamina, as for *T. kingii*. Only the slightly dilated lips of the sori could support it belonging to *C. atrovirens*, but the taxonomy remains debatable.

= *Trichomanes acrosorum* var. *alatum* Alderw., Bull. Jard. Bot. Buitenzorg, sér. 2, 11: 23 (1913). Type—New Guinea, near Leparei River, *s.d.*, *Janowski 52* (holotype: BO).

= *Cephalomanes densinervium auct. non* (Copel.) Copel.: H.Itô, Bot. Mag. (Tokyo) 67: 215 (1954).

Short description: Thick, short, and erect rhizomes with robust roots and clustered erect fronds; fronds well stipitate and once-pinnate, up to 40 cm long, linear-lanceolate; up to 6 sori per pinna, often distributed from the first third of the frond to the apex, sometimes on the whole frond, less often on the most apical pinnae (for the “*acranthum*” form), usually in proximal positions on the acroscopic margin and never reaching the apex of the pinnae, with truncate to slightly dilated lips.

Distribution: Ryukyu Islands (Japan) for the “*acranthum*” form, Philippines, eastern Borneo, Northern Sulawesi and Moluccas, Papua, New Guinea, Bismarck and Solomon Islands, Queensland and Lord Howe (Australia).

***Cephalomanes boryanum*** (Kunze) Bosch, Ned. Kruidk. Arch. 4: 351 (1859)

Based on *Trichomanes boryanum* Kunze, Farrnkräuter 1: 237 (1847) ≡ *Lacostea boryana* (Kunze) Prantl, Unters. Morph. Gefässkrypt.: 50 (1875) ≡ *Trichomanes javanicum* var. *boryanum* (Kunze) Fosberg, Amer. Fern J. 40: 136 (1950) ≡ *Cephalomanes atrovirens* subsp. *boryanum* (Kunze) K.Iwats., J. Jap. Bot. 66: 142 (1991). Type—Caroline Islands, Oualan, *s.d.*, *Dr. Mertens s.n.* (syntype: only drawing, Tab XCVII); Oualan, *s.d.*, *J. Dumont d‘Urville s.n.* (syntype: only drawing, L.I. Duperrey, Voy. Monde, Crypt.: 282 (1829) Pl. 38, Figure 2).

≡ *Trichomanes alatum* Bory, L.I.Duperrey, Voy. Monde, Crypt.: 282 (1829), non Sw., *nom. illeg*. ≡ *Cephalomanes alatum* C.Presl, Abh. Königl. Böhm. Ges. Wiss., ser. 5, 5: 334 (1848) ≡ *Trichomanes javanicum* var. *alatum* C.Chr., Index Filic.: 168 (1905). Based on the same type material as *T. boryanum* Kunze.

= *Cephalomanes australicum* Bosch, Ned. Kruidk. Arch. 5, 2: 139 (1861) ≡ *Trichomanes javanicum* var. *australicum* (Bosch) C.Chr., Index Filic.: 168 (1905) ≡ *Trichomanes australicum* (Bosch) Copel., Bull. Bernice P. Bishop Mus. 59: 27 (1929). Type—New Caledonia, Isle of Pines, *s.d.*, *Cuming 8* (lectotype: K!, designated by Iwatsuki and Ebihara [6]).

= *Cephalomanes wilkesii* Bosch, Ned. Kruidk. Arch. 5, 2: 140 (1861) ≡ *Trichomanes javanicum* var. *wilkesii* (Bosch) C.Chr., Index Filic.: 169 (1905). Type—Samoa or Fiji, 1838–1842, *Wilkes Expedition 24* (syntypes: GH00022275!, US00134632!). Note: Samoan Islands is written on the sheets, but Bosch also mentions the Fiji Islands (Ovalau) in his description.

Short description: Thick, short, and erect rhizomes with robust roots and clustered erect fronds; fronds well stipitate and once-pinnate, up to 40 cm long, ovate to lanceolate; up to 10 sori per pinna, usually on the most apical pinnae, less often distributed from the first third of the frond to the apex, usually in proximal positions on the acroscopic margin and rarely reaching the apex of the pinnae, with significantly dilated lips.

Distribution: Micronesia (Caroline Islands), Bismarck and Solomon Islands, Vanuatu, New Caledonia, Fiji, Samoa, French Polynesia.

## 5. Conclusions and Prospective Works

This study presents a taxonomic revision of *Cephalomanes javanicum* and *C. atrovirens*, demonstrating that the three varieties of the former and the two subspecies of the latter should be recognized as separate species. Consequently, the total number of recognized species in the genus increases to seven (excluding *C. singaporianum*). The taxonomic delimitation of these species is supported by comprehensive morphological investigations, particularly of sorus characteristics, in conjunction with geographic distribution patterns. Phylogenetic analyses further corroborate their status as distinct evolutionary lineages.

Further research should focus on expanding taxon sampling across the Indomalayan, Australasian, and Pacific regions, particularly including *C. crassum* and *C. densinervium*, as well as incorporating additional molecular markers. This will provide deeper insights into the evolutionary history, diversification, and biogeographical patterns of *Cephalomanes*, refining its classification and improving our understanding of its speciation processes.

## Figures and Tables

**Table 1:** Comparison of the three varieties of *C. javanicum* in addition to the two subspecies of *C. atrovirens*.

Groups or Taxa	*C. javanicum* var. *javanicum* (Figure 2)	*C. javanicum* var. *asplenioides* (Figure 3)	*C. javanicum* var. *sumatranum* (Figure 4)	*C. atrovirens* subsp. *atrovirens* (Figure 5)	*C. atrovirens* subsp. *boryanum* (Figure 6)
Number of sori per fertile pinna	1–9 (more often 3)	1–12 (more often 4–5)	1–3 (rarely more, up to 5)	1–6 (usually 3 or less than 5)	1–10 (more often 4–5)
Distribution of sori (see Figure 1)	Only acroscopic to distal side of pinna	Acroscopic to distal side, sometimes basiscopic distal portion	Only at the apex of pinna	Only at the base of acroscopic side of pinna	Often at the base, sometimes to the apex of acroscopic side of pinna
Sorus mouth and lips	Truncate to slightly dilated or flared	Usually truncate	Usually truncate	Truncate to slightly dilated or flared	Usually with significantly dilated lips
Lamina shape	Elliptic-linear to linear or narrowly lanceolate	Ovate-linear to linear-lanceolate	Elliptic-linear to linear or narrowly lanceolate	Linear-lanceolate	Ovate to lanceolate
Lamina base shape	Acute to obtuse	Obtuse to cuneate	Acute	Acute	Cuneate to truncate
Lamina length (cm)	4.5–30	5–25	6.5–20	7–26	9–24
Stipe length (cm)	1.5–19	1–17.5	2–10	2–13	2–13
Geographic distribution (see Figure 7)	East India to Java and Borneo (mostly in the western part)	Japan (Ryukyu Islands), and Taiwan to eastern Borneo and Solomon Islands	Hainan, northern Vietnam to Borneo and Sumatra	Japan (Ryukyu Islands), and Philippines to Queensland (Australia), and Solomon Islands	Micronesia, and Bismarck Islands to Polynesia

**Table 2:** Descriptive statistics computed for the morphological measurements of fronds and for the sorus data, which include the number of sori per pinna and their relative positions on the pinna (as detailed in the text).

Groups or Taxa	*C. javanicum* var. *javanicum*	*C. javanicum* var. *asplenioides*	*C. javanicum* var. *sumatranum*	*C. atrovirens* subsp. *atrovirens*	*C. atrovirens* subsp. *boryanum*	Test
Number of specimens measured	71	94	24	36	27	
Stipe length (cm)	6.33 (4.05) a	6.88 (3.35) a	**4.03 (1.75) b**	6.32 (3.31) a	**6.96 (2.80) a**	K-W
Lamina length (cm)	14.58 (5.35) a	15.45 (4.74) a	**11.00 (3.03) b**	15.93 (4.27) a	**16.90 (3.63) a**	G H
Lamina width (cm)	2.93 (0.75) a	3.21 (0.79) a	**2.31 (0.60) b**	2.60 (0.59) b	**3.61 (1.04) a**	K-W
Frond length (cm)	20.91 (8.75) a	22.33 (7.44) a	**15.03 (4.27) b**	22.26 (6.02) a	**23.86 (5.70) a**	K-W
Lamina/stipe ratio	0.40 (0.18) a	0.38 (0.15) a	0.46 (0.16) a	0.44 (0.22) a	0.41 (0.17) a	K-W
Number of fertile pinnae selected	168	132	87	69	53	
Number of sori per fertile pinna	3.11 (1.43) a	3.70 (1.75) a	2.21 (0.92) b	**2.17 (1.33) b**	**3.77 (1.84) a**	K-W
Relative position of the most proximal sorus	0.47 (0.02) a	0.68 (0.15) b	**0.82 (0.11) c**	0.35 (0.10) d	**0.33 (0.10) d**	K-W
Relative position of the most apical sorus	0.69 (0.15) a	0.86 (0.11) b	**0.88 (0.11) b**	**0.49 (0.14) c**	0.60 (0.16) c	K-W

The results are presented as means accompanied by standard deviations in parentheses. A distinction in letters following the values signifies a statistically significant difference (*p* < 0.05), as determined by a Bonferroni correction applied to non-parametric multiple comparison tests of means, as specified in the final column (K-W = Kruskal-Wallis; G H = Games-Howell). Values presented in bold denote the group or groups that exhibit the most pronounced characteristics for the trait under investigation.

**Table 3:** Genetic distances (here, the *p*-distance) of *rbc*L data between the specimens or accessions used for the phylogenetic analysis.

	2	3	4	5	6	7	8	9
1. *C. javanicum* var. *javanicum* Thailand	0.009	0.045	0.020	0.020	0.020	0.046	0.017	0.020
2. *C. javanicum* var. *javanicum* Borneo		0.051	0.024	0.024	0.024	0.051	0.022	0.024
3. *C. javanicum* var. *sumatranum* Hainan			0.040	0.038	0.040	0.008	0.037	0.040
4. *C. javanicum* var. *asplenioides* Taiwan1				0.001	0	0.041	0.008	0.011
5. *C. javanicum* var. *asplenioides* Taiwan2					0.002	0.039	0.008	0.010
6. *C. javanicum* var. *asplenioides* Japan						0.041	0.008	0.011
7. *C. atrovirens* subsp. *atrovirens* Japan							0.038	0.041
8. *C. atrovirens* subsp. *boryanum* Pohnpei								0.002
9. *C. atrovirens* subsp. *boryanum* Fiji								

## Data Availability

For molecular analyses, previously published sequences and newly acquired sequences are freely available on GenBank (“https://www.ncbi.nlm.nih.gov/genbank/”, accessed on 9 April 2025) as specified in Appendix A. All quantitative measurements are available on request.

## Appendix A

List of the selected specimens for the phylogeny with origin, GenBank accession number for *rbc*L sequence, and voucher information.

**Species**	**Origin**	**Herbarium**	**GenBank Number**	**Specimen Voucher**
*Cephalomanes javanicum* var. asplenioides	Taiwan (#1)	TNS	PV441474, this study	*Ebihara 031119-10*
*C. javanicum* var. asplenioides	Taiwan (#2)	P	PV368236, this study	*Knapp 3988*
*C. javanicum* var. asplenioides	Japan, Okinawa Pref.	TNS	AB574703	*TNS 759314*
*C. javanicum* var. javanicum	Thailand	TNS	PV441473, this study	*Iwatsuki 99H05*
*C. javanicum* var. javanicum	Borneo	MPU	Y09195	*Dubuisson ISEM H3001*
*C. javanicum* var. sumatranum	China, Hainan	SZG	PV368235, this study	*Chen 12715*
*C. atrovirens* subsp. a*trovirens*	Japan	TNS	LC484372	*TNS* *1176791*
*C. atrovirens* subsp. *boryanum*	Micronesia, Pohnpei	TI	AB257484	*Kokubo Ponape-12*
*C. atrovirens* subsp. *boryanum*	Fiji	TNS	AB257485	*Matsumoto 517*
*Callistopteris baldwinii*	Hawaii	PTBG	MT657789	*Wood 12395*
Ca. *polyantha*	French Polynesia, Moorea	UC	EU122962	*Nitta 068*
Ca. *apiifolia*	Taiwan	TNS	MH265125	*Matsumoto 0003-054*
*Abrodictyum rigidum*	Bolivia	UC	AY095108	*Kessler 11360*
*A. obscurum*	Japan	TNS	AB257480	*Ebihara 001118-06*
*A. flavofuscum*	New Caledonia	P	AY175804	*Munzinger 316*
*Trichomanes madagascariense*	Madagascar	P	KP153240	*Rouhan 367*
*T. osmundoides*	Guadeloupe	P	LC620556	*Chaussidon 23-2007*
*T. crispum*	Guadeloupe	P	LC620549	*Chaussidon 22-2007*
*Crepidomanes inopinatum*	Madagascar	P	AB257463	*Rakotondrainibe. 6261*
*Cr. parvulum*	Australia	TNS	AB479140	*Ohsawa 001201-03*
*Cr. thysanostomum*	Japan, Okinawa, Iriomote Isl.	TNS	AB083294	*Ebihara 001118-04*
*Vandenboschia speciosa*	France, Brittany	MPU	Y09201	*Dubuisson ISEM H401-410*
*V. striata*	Japan	TNS	OM160645	*Ebihara 991121-03*
*V. radicans*	Bolivia	UC, LPB	AF275650	*Kessler 11447*
*Didymoglossum reptans*	Brazil	BHCB	OQ162177	*Salino 13280*
*D. tahitense*	Japan	TNS	AB257498	*Ebihara 001119-01*
*D. cuspidatum*	Reunion	P	AF537122	*Dubuisson HR-1999-5*
*Polyphlebium vieillardii*	New Caledonia	TI	AB257471	*Ebihara 001220-02*
*P. venosum*	New Zealand	UC	AY175786	*Smith 2598*
*P. diaphanum*	Guadeloupe	P	KX913313	*Chaussidon 4-2007*
*Hymenophyllum inaequale*	Reunion	P	AY095112	*Dubuisson HR-1999-9 (P, F)*
*H. hirsutum*	Reunion	P	AY775407	*Dubuisson HR-1999-6*
*H. tunbrigense*	France, Brittany	MPU	Y09203	*Malengreau s.n. = Dubuisson ISEM H301*
*H. nephrophyllum*	New Zealand	TI, CHR	AB083290	*Ebihara 0011222-07*
